# Dietary Intake and Sources of Potassium in a Cross-Sectional Study of Australian Adults

**DOI:** 10.3390/nu11122996

**Published:** 2019-12-06

**Authors:** Kristy A. Bolton, Kathy Trieu, Mark Woodward, Caryl Nowson, Jacqui Webster, Elizabeth K. Dunford, Bruce Bolam, Carley Grimes

**Affiliations:** 1 School of Exercise and Nutrition Sciences, Deakin University, Geelong 3220, Australia; 2 The George Institute for Global Health, University of New South Wales, Camperdown, NSW 2050, Australia; ktrieu@georgeinstitute.org.au (K.T.); markw@georgeinstitute.org.au (M.W.); jwebster@georgeinstitute.org.au (J.W.); edunford@georgeinstitute.org.au (E.K.D.); 3 The George Institute for Global Health, University of Oxford, Oxford OX1 3QX, UK; 4 Institute of Physical Activity and Nutrition, Deakin University, Geelong 3220, Australia; caryl.nowson@deakin.edu.au (C.N.); carley.grimes@deakin.edu.au (C.G.); 5 Department of Nutrition, Gillings School of Public Health, The University of North Carolina, Chapel Hill, NC 27599, USA; 6 Department of Health and Human Services, Melbourne, VIC 3000, Australia; Bruce.Bolam@dhhs.vic.gov.au

**Keywords:** potassium consumption, potassium excretion, dietary assessment, urinary excretion, cardiovascular disease prevention, adults, purchasing origin, population prevention

## Abstract

A diet rich in potassium is important to reduce the risk of cardiovascular disease. This study assessed potassium intake; food sources of potassium (including NOVA level of processing, purchase origin of these foods); and sodium-to-potassium ratio (Na:K) in a cross-section of Australian adults. Data collection included 24-h urines (*n* = 338) and a 24-h diet recall (subsample *n* = 142). The mean (SD) age of participants was 41.2 (13.9) years and 56% were females. Mean potassium (95%CI) 24-h urinary excretion was 76.8 (73.0–80.5) mmol/day compared to 92.9 (86.6–99.1) by 24-h diet recall. Na:K was 1.9 (1.8–2.0) from the urine excretion and 1.4 (1.2–1.7) from diet recall. Foods contributing most to potassium were potatoes (8%), dairy milk (6%), dishes where cereal is the main ingredient (6%) and coffee/coffee substitutes (5%). Over half of potassium (56%) came from minimally processed foods, with 22% from processed and 22% from ultraprocessed foods. Almost two-thirds of potassium consumed was from foods purchased from food stores (58%), then food service sector (15%), and fresh food markets (13%). Overall, potassium levels were lower than recommended to reduce chronic disease risk. Multifaceted efforts are required for population-wide intervention—aimed at increasing fruit, vegetable, and other key sources of potassium intake; reducing consumption of processed foods; and working in supermarket/food service sector settings to improve the healthiness of foods available.

## 1. Introduction

The leading cause of death and disability in Australia is cardiovascular disease (CVD) [1]. A key modifiable risk factor for the development of CVD is diet. Characteristically, today’s Western diets have a decreased potassium intake (due to a reduced vegetable and fruit intake) and in parallel; an increased sodium intake via increased consumption of processed foods [2]. 

Low potassium intake is a risk factor for CVD [2]. In adults, the National Health and Medical Research Council (NHMRC) in Australia, recommends an adequate intake (AI) for potassium of 100 mmol/day (3800 mg/day) for men and 72 mmol/day (2800 mg/day) for women [3]. Additionally, the suggested dietary target (SDT) for potassium is 120 mmol/day (4700 mg/day potassium) which may help in the prevention of chronic disease [4].

Increasing intake of potassium has been suggested to reduce blood pressure, decrease CVD risk, have beneficial effects on bone-mineral density, and attenuate the negative consequences associated with consuming high amounts of sodium [2,5,6,7]. A recent meta-analysis revealed increasing dietary potassium has a protective effect against stroke and may reduce risk of CVD, recommending individuals to increase consumption of potassium-rich foods to prevent vascular diseases [8]. Key dietary sources of potassium are largely fruits and vegetables—with potatoes being the highest source of potassium from all foods [2]. Other key sources, according to data from the US National Health and Nutrition Examination Survey (NHANES), include milk, coffee, chicken and beef dishes and orange/grapefruit juices [2,6].

While there is increasing evidence of the health benefits of potassium, the majority of countries have implemented national strategies to lower salt intake exclusively. The key health consequence of a high dietary salt intake is high blood pressure [9,10] and Australia has committed to reducing the average salt intake by 30% by 2025, as per World Health Organization (WHO) recommendations [11]. Currently salt reduction strategies are being implemented in various countries to support individuals reducing their sodium intake [12,13]. However, potassium intake is often not a focus of these strategies; and therefore a missed opportunity to improve health. Potassium and sodium consumption should not be considered in isolation for their effects on health [2]. The interaction between potassium and sodium is subadditive; with both potassium and sodium having an effect on blood pressure [2]. This is why it is important to examine the sodium:potassium intake ratio (Na:K). An excess of sodium and a deficit in potassium increases the risk of hypertension and CVD [14,15]. Potassium intake should be at a level which will keep the Na:K close to 1.0 [16]. It has been demonstrated that high Na:K is more strongly related to CVD risk compared to sodium or potassium in isolation [2,17]. 

There is limited evidence using the objective measure of 24-h urine collections to assess potassium intake in Australians [18,19]. The aims of the current study were to assess potassium intake, as well as food sources of potassium (including level of processing, purchase origin of these foods); and the Na:K in a cross-section of Australian adults.

## 2. Materials and Methods

### 2.1. Participant Recruitment

Participants recruited for this study were intended to be reflective of the age and gender distribution of the adult population in the state of Victoria in Australia [20]. Participants were drawn from three sources—a previous study conducted in 2014 [19] (which used participants from the Victorian Health Monitor survey [21]); a random selection from the Victorian electoral roll; and orientation week at two Deakin University campuses (one urban, one rural; due to low response rates of individuals age 18–34 years old). Participant exclusion criteria were: not aged between 18–65 years old at the time of consent, currently undergoing chemotherapy and not living close to a Dorevitch Pathology Laboratory Centre. To participate, individuals provided written informed consent. Upon completion of the urine collection, a $20 supermarket voucher was offered.

### 2.2. Demographic and Anthropometric Data

Basic demographic information (i.e., date of birth, gender, postcode), anthropometry, medications and supplement use was collected via self-reported survey. Body mass index (BMI) was calculated from self-reported height and weight (weight (kg)/height (m^2^)) and participants were grouped according to WHO BMI classifications [22]. 

### 2.3. Collection of 24 h Urine Data

Urine was collected over a 24 h period. A timesheet, completed by the participant, included the date, start and finish times of urine collection, and whether any urine was missed (and the quantity of urine that was missed). Local Dorevitch Pathology centres (an Australian accredited commercial pathology service centre) examined urine volume, sodium and potassium for the 24-h samples using Ion Selective Electrode methodology; and creatinine concentrations were determined (using Jaffe, alkaline picrate, kinetic with black rate correction methodology) in a Siemens ADVIA 2400 autoanalyser. Urine collection times were standardised to a 24-h period. Standardised 24-h urine samples were excluded if: Females had a creatinine <4 mmol/24-h or outliers (>3SD from female mean; males with creatinine <6 mmol/24-h or outliers (>3SD from male mean); urine volume <500 mL; more than 1 reported void missing of >300 mL. The molecular weights of potassium (39 g/mmol), sodium (23 g/mmol), sodium chloride (58.5 g/mmol) were used to convert millimoles to milligrams.

### 2.4. Collection and Analysis of 24-h Dietary Recall Data

To examine the dietary sources of potassium, a single telephone administered 5-pass 24-h diet recall, taking approximately 20–30 min to complete, was conducted within two weeks of the urine collection. The day of collection depended on participant and staff availability. The aim was 200 diet recalls (100 males, 100 females) with a gender and age distribution reflective of the Victorian population, as detailed above. Most of the diet recalls related to dietary intake between Monday and Friday (79%) and the remainder on a Sunday (21%).

The 5-pass method used was based upon the 2011–12 National Nutrition and Physical Activity Survey: (i) Quick list, (ii) forgotten foods, (iii) time and occasion, (iv) detail cycle and (v) final probe [23]. A validated food model booklet was provided to participants to help them estimate portion sizes [24].

To determine the source of foods consumed, the question “Where did you get this/most of the ingredient for this (food name)?” [25] was asked. Response categories were based on the US NHANES) [25] but modified for the Australian context. 

Data were entered into FoodWorks V.8 (Xyris). Nutrient intakes were calculated using the Australian nutrient composition database AUSNUT (2011–2013) [26]. Potassium and sodium intakes were reported. The Schofield equation [27] was utilised to estimate basal metabolic rate (BMR) and the Goldberg method was utilised to identify under-reporters (i.e., ratio of Energy intake:estimated BMR (EI:estBMR)) with the appropriate cut-off value for the sample size (EI:estBMR < 1.49 for *n* = 200) [28]. Food items consumed were matched to an AUSNUT food code [26] and were used to determine the contribution of potassium from major and sub-major food groups. Major and sub-major food items were coded using the 2 digit, and 3 digit numeric classification codes, respectively [26]. The full list of food items can be accessed as a spreadsheet [29]. 

### 2.5. Comparison of Potassium Intakes to Dietary Guidelines

Potassium intake was based on 24-h diet recall values and compared to the NHMRC guidelines. Specifically, the group’s mean intake, by gender, was compared to the NHRMC’s Adequate Intake (AI) for potassium of 100 mmol/day (3800 mg/day) for men and 72 mmol/day (2800 mg/day) for women [3]. If the mean intake is above the AI a low prevalence of inadequate intakes can be assumed [3]. In addition, the group’s median intake was compared to the suggested dietary target (SDT) for potassium of 120 mmol/day (4700 mg/day potassium). This SDT reflects the median intake of a nutrient within the population that may help in the prevention of chronic disease [4]. 

### 2.6. Categorising the Level of Processing of Foods Using NOVA Classification System

The NOVA processing classification system [30] was applied to the food items in the Australian nutrient composition database AUSNUT database (2011–2013) as previously described [31]. Briefly, the NOVA processing classification system categorises foods into four groups based upon their level of processing [30]. This includes unprocessed and minimally processed (e.g., fresh/frozen fruit and vegetables, pasteurised milk), processed culinary ingredient (e.g., plant oils, animal fats), processed (e.g., canned vegetables/pulses, cheese) and ultraprocessed (e.g., chips, crisps, sweet/fatty/salty snacks, cereals, cakes).

### 2.7. Classifying Foods into Core and Discretionary

Individual foods were also classified as being a core or discretionary food as defined by the Australian Guide to Healthy Eating [32,33] using methods previously described [18]. Discretionary foods are those foods which are not a part of the five core food groups (vegetables; fruit; grain; lean meats, poultry, fish, eggs, tofu, nuts, seeds, legumes, beans; milk, yoghurt, cheese) and are not essential to the diet [33]. These foods are high in energy, saturated fat, added sugars, salt or alcohol and include foods such as sweet biscuits, cakes, pastries, processed meats, confectionery, fried foods, fatty and/or salty snacks, sugar-sweetened beverages [33].

### 2.8. Data Analysis

The Socioeconomic Index for Areas (SEIFA) Index of Relative Socioeconomic Advantage and Disadvantage, based on postal codes, was used to estimate the level of socioeconomic disadvantage [34]. SEIFA deciles were consolidated into quintiles for analysis.

Stata v15.0 (StataCorp LLC, Texas, USA) was used to analyse data. A post-stratification weight created using the age and gender distribution of the Victorian adult (18–65 years) population [20] was applied in Stata (pweight) due to the under-representation of younger aged males and females. All data presented were weighted; for unweighted data, please see supplementary data files. Descriptive statistics (mean (SD), mean (95%CI), median (IQR), *n* (%)) were calculated. Differences in urinary electrolyte excretion, dietary potassium, dietary Na:K and energy by sex were examined using an unpaired *t*-test. Differences in potassium, Na:K, energy by age category and level of socioeconomic disadvantage were determined using the svy:regress command in Stata. The population proportion method [35] was used to determine the contribution of potassium from the different food categories and each of the food origins (e.g., store, fresh food market, vending machines). A *p* value of <0.05 was considered statistically significant in all cases.

### 2.9. Ethical Approval

All subjects gave their informed consent for inclusion before they participated in the study. Ethics approval was obtained from the Faculty of Health Human Ethics Advisory Group, Deakin University (HEAG-H 71_2016).

## 3. Results

The number of individuals invited to the study was 6169 (*n* = 271 from the 2014 study; *n* = 5694 from the electoral roll, *n* = 204 from orientation week participants). Of the 345 urine samples analysed, seven samples met the exclusion criteria listed above and were removed from the analysis. Therefore the final sample size was 338 participants. The combined response rate for completed data was 7.5%. Of the 338 participants, 155 (65 males, 90 females) completed the 24-h dietary recall; however the final sample size was 142 participants after removing under-reporters (using Schofield/Goldberg cut offs *n* = 4); and participants without body weight (under-reporting could not be determined, *n* = 9). 

Demographic characteristics of adults with a complete urine collection, along with characteristics of the subgroup who also completed the diet recall is presented in Table 1. Compared to males, females were significantly younger and had a lower BMI (*p* < 0.05).

Regarding urinary electrolyte excretion (Table 2); mean potassium excretion was significantly higher for males compared to females (*p* < 0.001). There was no significant difference in Na:K by sex. The same significant differences by sex existed for the diet recall data for potassium (i.e., males had higher values); and no difference in Na:K. Among the sub-sample (*n* = 142) who had complete 24-h urine and recall data, compared to the diet recall data; urinary electrolyte data was lower for potassium and higher for Na:K (*p* < 0.05). To compare dietary potassium intake to the dietary guidelines [3,4], mean potassium was compared to the AI, and the median potassium compared to the SDT. The mean dietary potassium intake met the AI for males and females; but the median dietary potassium was much lower than the SDT of 120 mmol/day [4]. The younger age group (18–34 year olds) had a significantly higher urinary Na:K compared to older age group (55–65 year olds, *p* < 0.03). Individuals in quintile 1 of socioeconomic disadvantage (most disadvantaged) had a significantly lower urinary potassium excretion compared to all other quintiles (*p* < 0.003).

### Food Sources of Potassium

**Figure 1:**
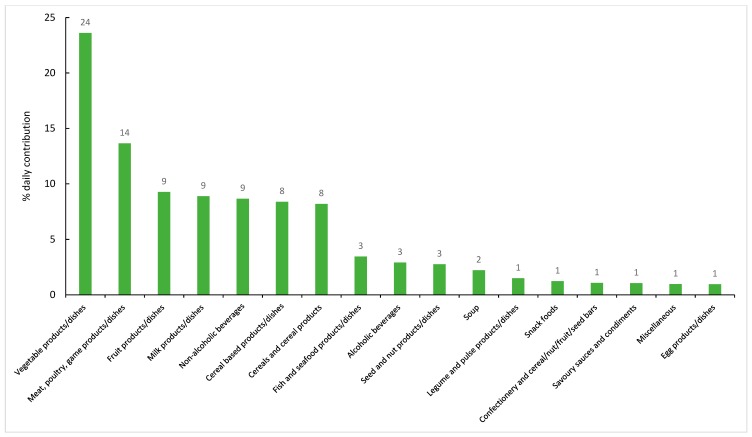
Contribution (%) of major food groups to dietary potassium (if contribution ≥1%) in a sample of Victorian adults aged 18–65 years (*n* = 142, weighted). Note: The total does not add to 100% as only data contributing to ≥1% of each source is presented.

**Figure 2:**
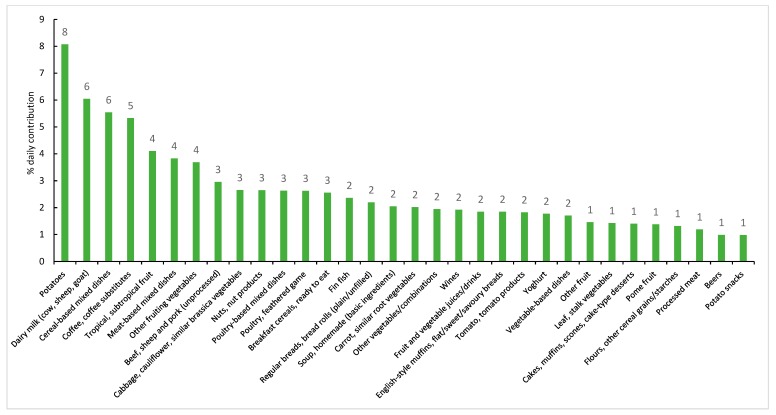
Contribution (%) of sub-major food groups to dietary potassium intake (if contribution ≥1%) in a sample of Victorian adults aged 18–65 years (*n* = 142, weighted). Note: The total does not add to 100% as only data contributing to ≥1% of each source is presented.

The contribution of food groups to dietary potassium are presented in Figure 1 (major food groups) and Figure 2 (sub-major food groups). The major contributing sources of dietary potassium were vegetable products and dishes (24%), meat, poultry and game dishes (14%), fruit products and dishes, milk products and dishes and non-alcoholic beverages (9% each). 

**Figure 3:**
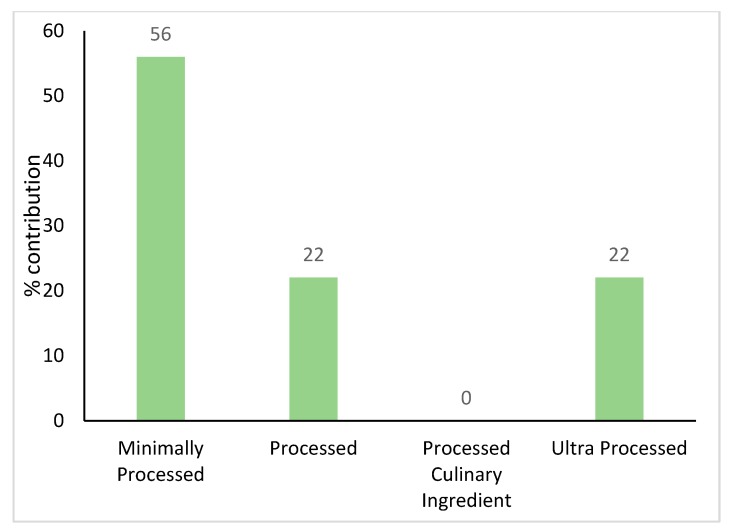
Daily contribution (%) to potassium by level of food processing in a sample of Victorian adults aged 18–65 years (*n* = 142, weighted).

Regarding the sub-major food groups (Figure 2); the top five sources of potassium intake were potatoes (8%), dairy milk (6%), dishes where cereal is the main ingredient (6%, e.g., pizza, sandwiches, burgers, savoury pasta, rice/noodle dishes), coffee and coffee substitutes (5%) and tropical and subtropical fruits (5%). When foods were categorised as either core or discretionary, 82% of all potassium came from core foods. When foods were categorised by level of food processing, the largest contributor to potassium intake was the minimally processed foods (Figure 3).

**Figure 4:**
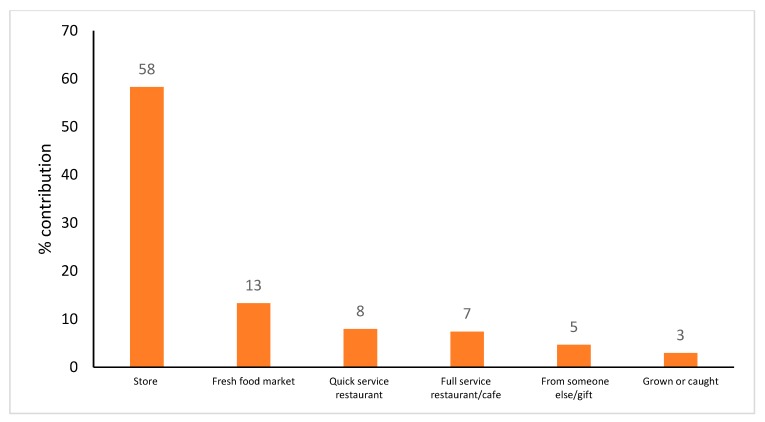
Purchase origin of potassium (if contribution ≥1%) in a sample of Victorian adults aged 18–65 years (*n* = 142, weighted). Note: store includes grocery/supermarket, convenience store, specialty; fresh food market includes the butcher, local/farmers/fruit and vegetables markets, green grocers; full-service restaurant includes sit down restaurant, cafe; quick service restaurant includes fast food chains, take-away, delivery. The total does not add to 100% as only data contributing to ≥1% of each source is presented.

Just over half (58%) of all potassium consumed was derived from foods originating from food stores (e.g., grocery/supermarket/convenience) (Figure 4). The remainder came from foods sourced at fresh food markets (e.g., butcher, local/farmers/fruit vegetable market, greengrocer) (13%), quick service restaurants/takeaways (8%), and full service restaurants (7%). Note that 3% of potassium consumed was missing the source location. 

## 4. Discussion

This population of Victorian adults in 2016/17 was consuming less than the SDT of potassium to prevent the risk of chronic disease. The mean urinary and dietary Na:K was higher than recommendations which is of concern as it suggests potassium intake is less than optimal; and sodium intake is higher than recommended. This may influence chronic disease risk and the development of hypertension, CVD and other non-communicable diseases [14]. Urinary Na:K was significantly higher in the younger compared to the older age groups. Urinary potassium was significantly lower in the most socioeconomically disadvantaged quintile of individuals. The main dietary sources of potassium were potatoes, dairy milk, dishes where cereal is the main ingredient and coffee/coffee substitutes. Ultraprocessed foods accounted for over a fifth of foods consumed. Almost two-thirds of potassium consumed was from foods purchased from food stores. Collectively, these results highlight the need to create awareness of increasing potassium intake by reducing intake of ultraprocessed foods; and increasing intake from minimally processed and core foods. 

### 4.1. Potassium

At the group level, participants in the current study were not consuming enough potassium to reduce risk of chronic disease. Potassium intake was higher in males compared to females; however, this could be due to the significantly larger amount of food (energy) males consumed within a 24-h period. Urinary potassium excretion was lower in individuals in the most socioeconomically disadvantaged quintile. Similarly, previous studies have reported lower equivalent household expenditure to be associated with lower potassium intake (and higher Na:K) in Japanese [38]; lower neighbourhood socioeconomic status to be associated with lower urinary potassium in women only [39]; low-skill workers to have lower potassium excretion compared to top managerial positions in Italy [40] and potassium excretion to be lower by education and income levels in New York City [41]. Potassium excretion has been correlated with fruit and vegetable intake [41] and in Australia, lower socioeconomic position was associated with lower diet quality and poorer nutrient intakes highlighting the importance of social determinants on diet quality [42]. 

A recent systematic review and meta-analysis revealed the beneficial effect of increasing potassium to reduce blood pressure in adults, and an inverse relationship between potassium intake and risk of stroke in adults [10]. Potassium estimates were lower in the urinary excretion data compared to the 24-h diet recall data, which was expected [43] and likely reflects the non-urinary losses of potassium lost via the faeces [44]. It has been reported that most of ingested potassium is excreted in urine, however, 10–20% can be excreted in faeces and sweat [45,46]. It is difficult to estimate the amount of potassium lost via non-urinary pathways, as it can depend on the diet, with a higher intake of fibre resulting in a higher loss of potassium in the faeces (IOM 2005) [45,47]. 

### 4.2. Na:K

The Na:K should ideally be 1.0 [16]. In this study, the Na:K by urine excretion was 1.9; and consequently this increases an individual’s risk of CVD [14,15]. Na:K > 1 have also been reported in other adult populations in Australia [18,19,48] and the US [49,50]. Examination of nationally representative data in 2011–2012 from NHANES revealed that only approximately 10% of US adults have a Na:K consistent with WHO guidelines and therefore may have reduced risk of CVD-related mortality [49]. The finding in this study that males had a higher potassium intake (via urinary excretion and dietary intake) compared to females is also similar to previous studies in Australia [48], US [50], UK [51], France [52] and Greece [43]. This disparity may be due to males consuming more overall energy per day. Population-wide promotion of high potassium-containing unprocessed foods which will have a favourable Na:K is required to shift Na:K to 1.0.

### 4.3. Sources of Dietary Potassium

The five major food sources of dietary potassium in this sample of Victorian adults included vegetable products and dishes; meat, poultry and game dishes; fruit products and dishes; milk products and dishes and non-alcoholic beverages. When categorised by sub-major food groups, the largest contributors of potassium were potatoes, dairy milk, dishes where cereal is the main ingredient, and coffee and coffee substitutes. Similarly, other recent Australian studies have also identified that vegetable produces/dishes (in particular, potatoes), breads and cereals, milk products/dishes and meat products/dishes are key contributors of potassium intake [18,48,53,54]. Potato as a key source of potassium is a common finding [18,48,53], however whilst other fruits and vegetables are also good sources of potassium, these are generally not featured in the same proportion as potato and represent an opportunity to improve food choices [48]. Additionally, cooking methods of potatoes such as cooking with fat, frying and adding salt; may influence its nutritional value in the diet [48,53]. Other opportunities to increase potassium intake could be to substitute commonly eaten white bread with wholemeal bread; as wholemeal bread can contain up to 70% more potassium [53].

### 4.4. Level of Processing of Foods in Relation to Potassium Intake

When foods were categorised as either core or discretionary, 82% of all potassium came from core foods. This is similar to data reported in Victorian primary schoolchildren [18]. This is encouraging given *The Australian Guide to Healthy Eating* recommends consumption of the five core food groups and limiting discretionary food choices [33]; however, this still equates to approximately a fifth of daily potassium originating from discretionary foods. Current diets are heavily reliant on packaged and processed foods [55]; with ultraprocessed foods dominating Western diets [56,57,58,59,60,61,62]. When foods were categorised by level of food processing, just over half of potassium intake was from minimally processed foods (56%); and just over a fifth from ultraprocessed foods. The consumption of ultraprocessed foods and increased risk of developing diet-induced chronic disease has been previously reported [59]. Decreasing dietary intake of ultraprocessed foods could not only effectively improve diet quality [62] but also reduce risk of diet-induced chronic disease. Replacing ultraprocessed food items with minimally processed foods is recommended; however, the population must have the knowledge and cooking skills to support this. This could also have an added impact of reducing sodium consumption [18,48] and improving Na:K. 

Information on the purchase origin of potassium and sodium are required in order to develop and implement contextually relevant and setting-specific strategies to create supportive environments and encourage increased consumption of potassium and reduce sodium intake which will contribute to a reduction in CVD. In the current study foods containing dietary potassium originated primarily from supermarkets/grocery stores (58%); with the next leading sources being fresh food markets (13%), and food service sector (15% including restaurants, cafes, fast food and convenience stores). The opportunity to reach the wider population via these settings with strategies to improve potassium consumption in conjunction with reducing sodium consumption should be taken. 

### 4.5. Implications

Understanding current dietary intake is crucial for informing the design of strategies to help individuals meet nutrient recommendations within energy needs [63] given the globally low consumption levels of potassium, and the potential health benefits on blood pressure and stroke [10]. Increasing potassium intake in the diet could be a cost effective measure to reduce the burden of morbidity and mortality from non-communicable diseases [10]. Consumers need help making healthy sustainable choices in order to achieve dietary guidelines [64]. The current food environment provides a multitude of food choices with conflicting messages and competing concerns; alongside consumer demands for taste, convenience and cost [64]. Identifying major food sources of potassium and communicating these at a population level in dietary education messages; along with creating supportive environments together is important to support individuals to meet guidelines for daily consumption. Focusing not only on potassium as a nutrient in isolation, but as part of whole foods; will make dietary messages easier to understand and achieve. Additionally, emphasising not only higher fruit and vegetable intake to achieve adequate potassium consumption, but considering other sources of potassium such as wholegrains, nuts, seeds and low fat dairy is recommended [53]. Tackling low potassium levels, and lack of compliance to dietary guidelines can only be achieved via a multi-pronged approach including consumers, food industry and policy makers [64]. 

### 4.6. Strengths and Limitations

Key strengths of this study include using the gold standard, objective measure of potassium excretion (24-h urine collection); validated methodology such as the diet recall [25,32]; food classification systems [26] and the NOVA level of processing classification system [65]. 

However, there are some study limitations. This study was conducted in a relatively large sample of Victorian adults from three pools, however there was an overall low response rate. To adjust for this, data were weighted by age and gender to be reflective of the distribution with the Victorian population. However, caution should be taken in making broad generalisations. Additionally, some participants may be motivated to participate due to an interest in health. A more comprehensive analysis by level of socioeconomic disadvantage was not possible due to low sample sizes in some quintiles of socioeconomic disadvantage. Recall and social desirability bias may influence the 24-h diet recall [66]. Under reporting whereby discretionary or ultraprocessed foods could be underestimated may also occur [58]. The singular 24-h diet recall, and 24-h urine collection collected may not be reflective of participants’ usual dietary intake or eating pattern [48]. However a single collection is adequate to examine the diet of large populations [67] and reduce a participants’ burden. The data in this study were not adjusted for non-urinary losses [5,10] as quantifying non-urinary loss is difficult and can range from 10–20% [45,46].

Future research should consider collecting the actual eating location along with the origin of purchase; as this information will help interpret an individuals’ interaction with the food environment they purchase from [67]. Despite these limitations, the data collected from the sample population in this study are useful to estimate group means of potassium intake; food sources and purchasing origin of food sources contributing daily potassium intake among Victorian adults.

## 5. Conclusions

In this Victorian sample of adults, not enough potassium was being consumed which may increase the risk of CVD-related mortality. This study has revealed leverage points for population-wide intervention—the development of strategies aimed at increasing fruit, vegetable, and other key sources of potassium; reducing consumption of discretionary and ultraprocessed foods; and working in supermarket/food service sector settings to improve the healthiness of foods available.

## Figures and Tables

**Table 1:** Demographic characteristics of a sample of Victorian adults aged 18–65 years (weighted).

	Proportion (%) or Median			
	Total *n* = 338	Males *n* = 148 (49%)	Females *n* = 190 (51%)	Victorian Population (%)49% Males, 51% Females ^a^
Age (years) *	41.2 (13.9)	41.0 (13.4)	41.4 (14.3)	37 ^a^
Age group (years)				
18–34	38	39	38	29 ^b^
35–54	42	42	42	26
55–65	20	20	20	11
BMI *	24.6 (4.1)	25.3 (3.5)	24.0 (4.5)	
BMI category				
Underweight	3	2	5	2 ^c^
Healthy weight	53	47	59	38
Overweight	35	42	28	31
Obese	9	10	9	19
Socioeconomic disadvantage (quintiles)				
1st quintile (greatest disadvantage)	6.3	2.9	9.5	
2nd quintile	6.7	7.0	6.4	
3rd quintile	9.2	9.5	8.8	
4th quintile	34.8	39.8	29.9	
5th quintile (least disadvantage)	43.1	40.8	45.3	

* Mean (SD) ^a^: data taken from Australian census and represents the median [20]; ^b^: data taken from ABS 2017 [36]. Note this statistic includes 15–24 year olds living in Victoria; ^c^: data from Victorian Population Health Survey 2016 (note proportion does not add to 100% due to responses such as “don’t know” or “refused” [37].

**Table 2:** Urinary electrolyte excretion and dietary intake in a sample of Victorian adults aged 18–65 years (weighted).

Measure	Total	Age Group (Years)			*p* Value	Males	Females	*p* Value	Socioeconomic Disadvantage (Quintiles)					*p* Value
**Urinary Data**	*n* = 338	18–34 *n* = 63	35–54 *n* = 155	55–65 *n* = 120		Total *n* = 148	Total *n* = 190		1 *n* = 23	2 *n* = 21	3 *n* = 40	4 *n* = 104	5 *n* = 150	
Urinary K (mmol/24-h) Mean (95%CI)	76.8 (73.0–80.5)	74.4 (66.5–82.3)	78.6 (73.7–83.4)	77.7 (73.1–82.2)	0.67	88.4 (82.3–94.4)	65.5 (62.6–68.6)	<0.001	61.0 ^b^ (54.1–68.0)	80.5 (67.0–94.0)	74.6 (64.9–84.3)	77.5 (70.6–84.3)	78.4 (72.7–84.0)	<0.002
Urinary K (mmol/24-h) Median (IQR)	71.5 (55.9–91.3)	68.1 (55.6–89.0)	73.7 (58.2–94.6)	73.9 (60.0–89.0)		86.4 (64.8–105.4)	64.5 (49.2–79.9)		55.6 (49.5–73.6)	77.9 (59.6–96.9)	65.0 (58.6–85.2)	77.0 (57.2–90.1)	73.3 (58.3–100.9)	
Urinary Na:K (mmol/24-h) Mean (95%CI)	1.9 (1.8–2.0)	2.0 ^a^ (1.8–2.3)	1.8 (1.7–2.0)	1.7 (1.6–1.9)	0.09	2.0 (1.8–2.1)	1.8 (1.7–1.9)	0.16	2.3 (1.8–2.8)	1.8 (1.5–2.1)	2.0 (1.7–2.3)	2.0 (1.8–2.2)	1.8 (1.6–1.9)	0.13
**Diet Recall Data**	Total *n* = 142	18–34 *n* = 28	35–54 *n* = 79	55–65 *n* = 35	*p* value	Total *n* = 148	Total *n* = 190	*p* value	1 *n* = 11	2 *n* = 7	3 *n* = 21	4 *n* = 31	5 *n* = 72	*p* value
K diet recall (mmol/24-h) Mean (95%CI)	92.9 (86.6–99.1)	85.4 (73.7–97.2)	95.2 (88.3–102.1)	102.6 (89.7–115.6)	0.14	100.2 (88.5–111.8)	85.8 (79.4–92.3)	<0.04	97.1 (71.1–123.2)	76.0 (52.5–99.5)	96.4 (85.7–107.0)	95.1 (83.2–107.0)	91.9 (82.7–101.0)	0.62
K diet recall (mmol/24-h) Median (IQR)	85.1 (70.5–111.0)	80.0 (70.5–103.6)	86.0 (71.0–118.5)	103.2 (68.9–126.7)		102.9 (70.5–120.9)	81.2 (68.3–101.8)		86.0 (69.9–104.9)	63.5 (59.2–80.3)	94.1 (81.3–109.4)	86.8 (80.0–108.8)	82.5 (70.5–114.5)	
NA:K diet (mmol/24-h) Mean (95%CI)	1.4 (1.2–1.7)	1.6 (1.1–2.2)	1.3 (1.2–1.5)	1.1 (0.9–1.3)	0.12	1.5 (1.0–2.0)	1.3 (1.2–1.5)	0.45	1.3 (1.0–1.5)	1.7 (1.2–2.3)	1.4 (1.1–1.7)	1.3 (0.9–1.6)	1.5 (1.1–1.9)	0.61
Energy (kJ/day) diet recall Mean (95%CI)	10,043.2 (9457.4–10,629.1)	9973.6 (8662.8–11,284.4)	10,223.9 (9653.0–10,794.7)	9791.2 (8876.1–10,706.4)	0.72	10,827.6 (9817.1–11,838.1)	9284.9 (8708.0–9861.8)	<0.01	9712.1 (8328–11,095.9)	10,327.6 (7920.4–12,734.8)	10,478.8 (9711.7–11,245.9)	10,136.1 (8257.4–12,014.7)	9919.5 (9343.7–10,495.3)	0.80
Potassium density (g potassium/MJ energy)	0.37 (0.35–0.39)	0.35 (0.30–0.40)	0.37 (0.35–0.39)	0.41 (0.37–0.45)	0.07	0.37 (0.33–0.41)	0.37 (0.35–0.40)	0.88	0.38 (0.32–0.44)	0.30 (0.23–0.37)	0.36 (0.32–0.40)	0.39 (0.34–0.44)	0.37 (0.33–0.41)	0.41

^a^ Statistically significant compared to 55–65 year old age category (*p* < 0.03); ^b^ statistically significant to all other quintiles (*p* < 0.003). Note: quintile 1 of socioeconomic disadvantage is the greatest disadvantaged; and quintile 5 is the least disadvantaged.

## Supplementary Materials

The following are available online at https://www.mdpi.com/2072-6643/11/12/2996/s1, Figure S1: Contribution (%) of potassium (unweighted) from major food groups (if contribution ≥1%) in a sample of Victorian adults aged 18–65 years (*n* = 142) Figure S2: Contribution (%) of potassium (unweighted) from sub-major food groups (if contribution ≥1%) in a sample of Victorian adults aged 18–65 years (*n* = 142), Figure S3: Daily contribution (%) of potassium (unweighted) by level of food processing in a sample of Victorian adults aged 18–65 years (*n* = 142), Figure S4: Purchase origin of potassium (if contribution ≥1%) in a sample of Victorian adults aged 18–65 years (*n* = 142, weighted).
