# Supplementary material for: Dietary Intake and Sources of Potassium in a Cross-Sectional Study of Australian Adults
Source: Nutrients. 2019 Dec 6;11(12):2996. doi: 10.3390/nu11122996 (PMC6949933; doi:10.3390/nu11122996)
Supplement: Supplementary file 1 [file nutrients-11-02996-s001.pdf]

Supplementary figures of unweighted data

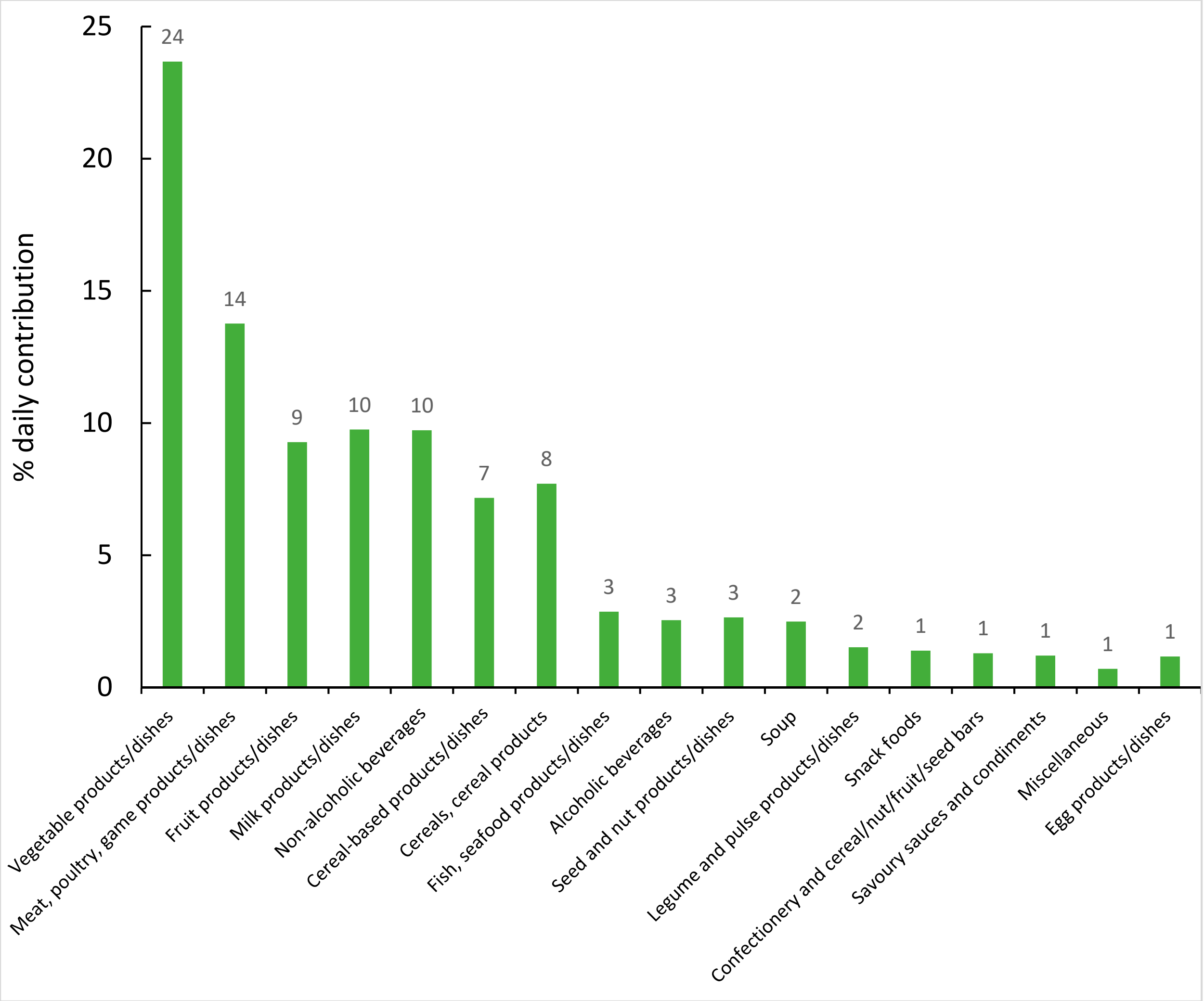

Supplementary figure 1: Contribution (%) of potassium (unweighted) from major food groups (if contribution ≥1%) in a sample of Victorian adults aged 18-65 years (n=142)

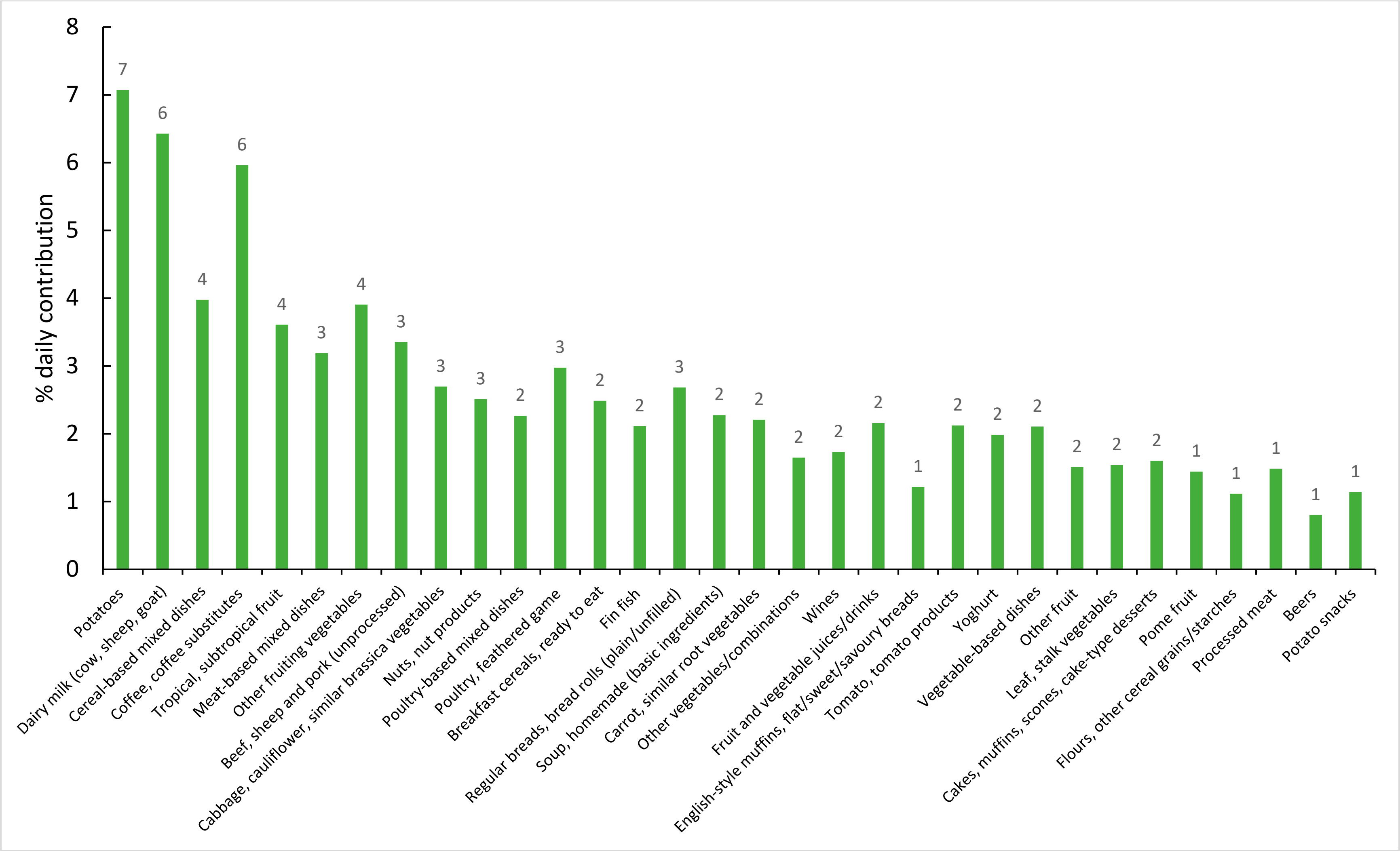
% daily contribution
0
1
2
3
4
5
6
7
8
Potatoes
Dairy milk (cow, sheep, goat)
Cereal-based mixed dishes
Coffee, coffee substitutes
Tropical, subtropical fruit
Meat-based mixed dishes
Other fruiting vegetables
Beef, sheep and pork (unprocessed)
Cabbage, cauliflower, similar brassica vegetables
Nuts, nut products
Poultry-based mixed dishes
Poultry, feathered game
Breakfast cereals, ready to eat
Fin fish
Regular breads, bread rolls (plain/unfilled)
Soup, homemade (basic ingredients)
Carrot, similar root vegetables
Other vegetables/combinations
Wines
Fruit and vegetable juices/drinks
English-style muffins, flat/sweet/savoury breads
Tomato, tomato products
Yoghurt
Vegetable-based dishes
Other fruit
Leaf, stalk vegetables
Cakes, muffins, scones, cake-type desserts
Pome fruit
Flours, other cereal grains/starches
Processed meat
Beers
Potato snacks
7
6
4
6
4
3
4
3
3
3
2
3
2
2
3
2
2
2
2
2
1
2
2
2
2
2
2
1
1
1
1
1

Supplementary figure 2: Contribution (%) of potassium (unweighted) from sub-major food groups (if contribution ≥1%) in a sample of Victorian adults aged 18-65 years (n=142)

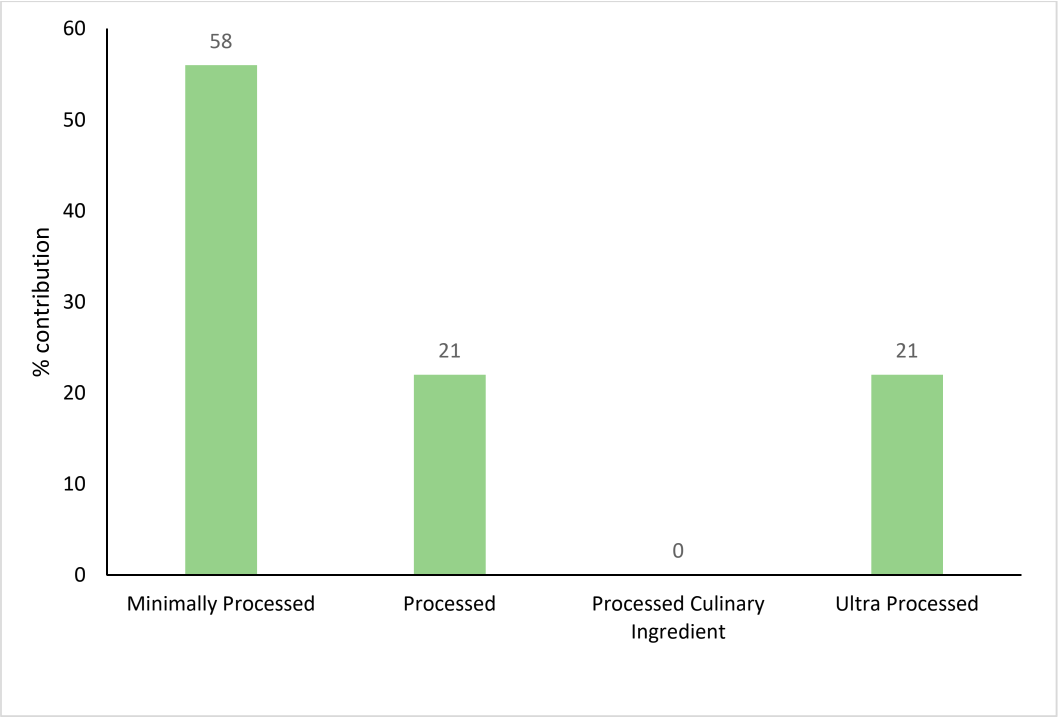

Figure 3: Daily contribution (%) of potassium (unweighted) by level of food processing in a sample of Victorian adults aged 18-65 years (n=142)

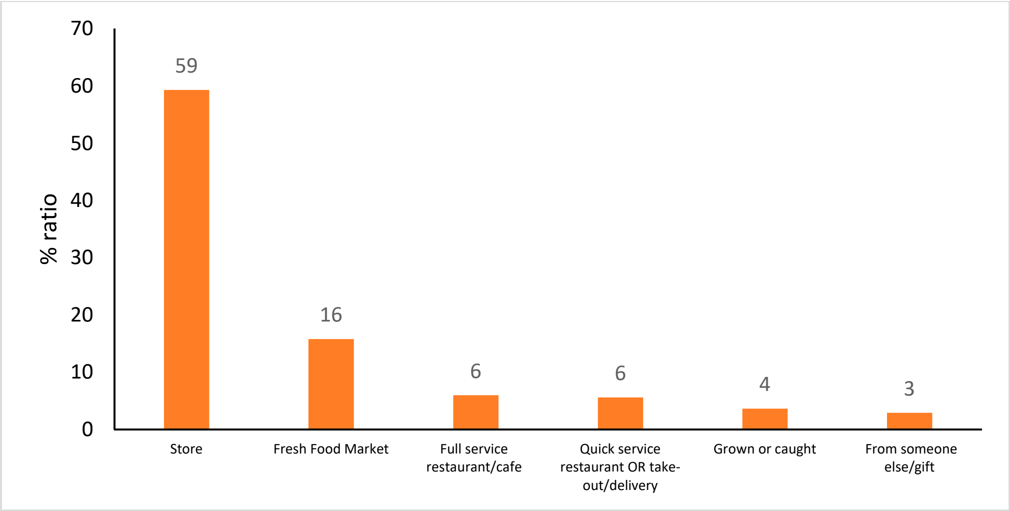

Supplementary Figure 4: Purchase origin of potassium (if contribution ≥1%) in a sample of Victorian adults aged 18-65 years (n=142, weighted)

Note: store includes grocery/supermarket, convenience store, speciality; fresh food market includes the butcher, local/farmers/fruit and vegetables markets, green grocers; full service restaurant includes sit down restaurant, cafe; quick service restaurant includes fast food chains, take-away, delivery.
